# Multi-omics analysis identifies the unique high-FDCSP basal cells in triple-negative breast cancer

**DOI:** 10.3389/ebm.2025.10632

**Published:** 2025-09-25

**Authors:** Xinya Lu, Zhen Chen, Ying Shao

**Affiliations:** ^1^ Department of Surgical Pathology, Women’s Hospital, School of Medicine, Zhejiang University, Hangzhou, Zhejiang, China; ^2^ Department of Surgical Pathology, The First Affiliated Hospital of Medical School of Zhejiang University, Hangzhou, Zhejiang, China

**Keywords:** FDCSP, TNBC, multi-omics, TGFβ1-EGFR, TME

## Abstract

Follicular dendritic cell secreted protein (FDCSP) is highly expressed in various cancers and has been implicated in tumor migration and invasion, yet its role in triple-negative breast cancer (TNBC) remains poorly understood. Our findings revealed that FDCSP expression was significantly elevated in TNBC compared to normal breast tissue, whereas its expression was significantly reduced in non-TNBC. In TNBC, high FDCSP expression was associated with an increased mutation rate of TP53 and influenced the infiltration of B cells and macrophages. Single-cell transcriptome analysis demonstrated that FDCSP was predominantly highly expressed in basal cells but exhibited low expression in luminal epithelial cells. This observation was further corroborated by spatial transcriptome (ST) analysis. Immunohistochemistry (IHC) assay also confirmed the distinct expression patterns of FDCSP. Cell-cell interaction and receptor-ligand pair analyses indicated that macrophages could interact with the receptor epidermal growth factor receptor (EGFR) in FDCSP highly expressed basal cells by secreting transforming growth factor-β1 (TGF-β1). Then, the co-localization of FDCSP and EGFR in TNBC basal cells was verified by IHC and immunofluorescence (IF) assay. Additionally, we discovered that FDCSP possesses strong predictive capabilities for distinguishing between responders and non-responders to Immune checkpoint blockade (ICB) treatment. Finally, leveraging the CARE database, we identified 14 potential FDCSP-related target drugs. These findings highlight the unique expression pattern of FDCSP in breast cancer, revealing FDCSP as a promising target for therapeutic strategies in TNBC.

## Graphical Abstract

**Figure:**
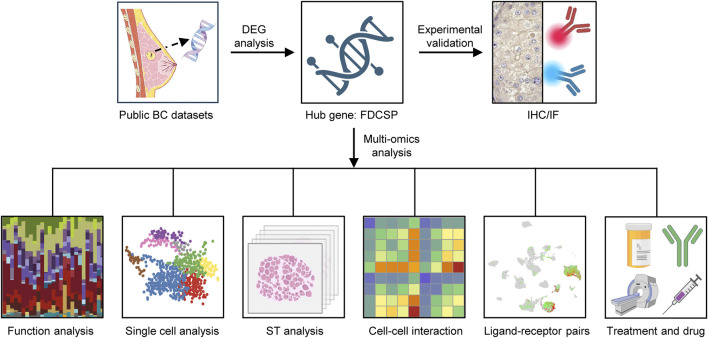
This study outlines its core methodology as follows. Initially, transcriptome data related to breast cancer were collected from public datasets, including TCGA and GEO, for differential expression analysis. FDCSP was identified as the key gene, based on which multi-omics analyses were conducted. These included function analysis, single-cell transcriptomic profiling, ST analysis, cell-cell interaction assessment, and ligand-receptor pair identification. In addition, the efficacy of ICB treatment and targeted drugs for FDCSP were predicted. Concurrently, IHC and IF assays were employed to validate the findings. In summary, this integrative approach enables a comprehensive understanding of the distinct role of FDCSP in TNBC from multiple analytical perspectives.

## Impact statement

This study aimed to identify key factors driving malignant progression in TNBC. To achieve this, we employed a multi-omics approach to comprehensively analyze the difference between TNBC and non-TNBC. Our findings revealed that FDCSP expression was significantly elevated in TNBC compared to normal breast tissue, whereas its expression was significantly reduced in non-TNBC. FDCSP was predominantly highly expressed in basal cells of TNBC and this observation was corroborated by spatial transcriptome analysis. Furthermore, macrophages could interact with the receptor EGFR in FDCSP highly expressed basal cells by secreting TGF-β1. FDCSP also demonstrates robust predictive value in discriminating between responders and non-responders to immune checkpoint blockade therapy. Based on these findings, our study highlights the potential of FDCSP as a therapeutic target and provides novel insights into targeted FDCSP-based strategies for breast cancer treatment.

## Introduction

According to GLOBOCAN 2022, breast cancer (BC) remains the most frequently diagnosed cancer and the leading cause of cancer-related mortality among women [1]. Breast cancer is classified into distinct subtypes based on the expression of estrogen receptor (ER), progesterone receptor (PR), and human epidermal growth factor receptor 2 (HER2). These subtypes include triple-negative breast cancer (TNBC), luminal (ER/PR-positive), and HER2-overexpressing breast cancer [2]. TNBC accounts for 15–20% of all breast cancer cases and is more prevalent among younger women under the age of 40. Compared to other subtypes, TNBC is associated with a mortality rate of up to 40%, a distant metastasis rate of 46%, and a recurrence rate of up to 25% within 5 years of diagnosis [3, 4]. Due to the absence of targetable receptors, both endocrine therapy and conventional targeted therapies are ineffective in treating TNBC. Although advancements have been made in conventional chemotherapy and neoadjuvant immunotherapy, a subset of patients continues to exhibit poor treatment responses and a high risk of recurrence or metastasis [5, 6].

The occurrence and progression of breast cancer are influenced by numerous factors, among which the tumor microenvironment (TME) plays a pivotal role. As the “soil” for cancer cell growth, the TME’s critical importance has been well-documented in numerous studies. The TME comprises tumor cells, stromal cells, infiltrating immune cells, endothelial cells, the extracellular matrix, and a variety of signaling molecules. The composition and dynamics of the TME significantly impact breast cancer progression [7], metastasis [8], anti-tumor immune responses [9], and therapeutic outcomes [10]. Therefore, an in-depth study of TME in breast cancer, especially TNBC, is necessary to improve the prognosis of patients.

Follicular dendritic cell secreted protein (FDCSP), also known as c4orf7, is a small secreted protein originally identified in follicular dendritic cells (FDCs). FDCSP is a unique secreted peptide with a distinct expression pattern in the immune system and exhibits specific binding affinity to activated B cells. FDCSP also has been reported to be highly expressed in several cancers, including ovarian cancer [11], head and neck squamous carcinoma (HNSC) [12], renal cell carcinoma (RCC) [13], and lung adenocarcinoma [14]. It has been proposed as a prognostic marker for HNSC and RCC and is thought to promote tumor metastasis by enhancing the migration and invasion of cancer cells [11]. Despite its established role in other malignancies, the expression and functional significance of FDCSP in breast cancer remain poorly understood and underexplored.

The aim of this study was to explore the expression patterns and functional role of FDCSP in TNBC. Through comprehensive analysis of breast cancer datasets, we discovered that FDCSP is specifically and highly expressed in TNBC epithelial cells, while its expression is nearly absent in non-TNBC epithelial cells compared to normal breast tissue. Further investigation revealed that FDCSP is associated with the TP53 mutation rate and macrophage infiltration. Specifically, macrophages were found to interact with the epidermal growth factor receptor (EGFR) on high-FDCSP basal cells in TNBC by secreting transforming growth factor-β1 (TGF-β1). Furthermore, FDCSP demonstrates robust predictive value in discriminating between responders and non-responders to immune checkpoint blockade (ICB) therapy. Based on these findings, our study highlights the potential of FDCSP as a therapeutic target and provides novel insights into targeted FDCSP-based strategies for breast cancer treatment.

## Materials and methods

### Data collection

The samples used in this study were obtained from publicly available datasets. RNA sequencing (RNA-seq) data for breast cancer were retrieved from The Cancer Genome Atlas (TCGA) database^1^ and the Gene Expression Omnibus (GEO) database,^2^ including datasets GSE76275, and GSE21653. For single-cell RNA sequencing (scRNA-seq) analysis, raw data from the GSE161529 dataset were downloaded from GEO. Spatial transcriptomic (ST) data were obtained from 10X Genomics^3^ and a publicly available study [15].

### Identification of differentially expressed genes

RNA-seq data from the TCGA-BRCA cohort and GSE76275 dataset were used for differential expression analysis. Principal Component Analysis (PCA) was employed to assess data distribution and identify potential batch effects. Differential gene expression analysis was performed using the R package limma, with adjustments for multiple hypothesis testing using the Benjamini-Hochberg false discovery rate (FDR) method. Genes with an adjusted *P-value* <0.05 and a fold change >1 or <−1 were classified as differentially expressed genes (DEGs) and selected for further analysis. Visualization of gene expression patterns was achieved using volcano plots and box plots generated with the ggplot2 R package. Venn diagrams were created using the Jvenn online tool.^4^ Gene expression heatmaps were constructed using the pheatmap R package. Functional enrichment analyses, including Gene Ontology (GO) and Kyoto Encyclopedia of Genes and Genomes (KEGG), were conducted using the clusterProfiler R package. GO and KEGG terms with an adjusted *P*-value <0.05 were considered statistically significant, and results were visualized using ggplot2. Protein-protein interaction (PPI) networks were analyzed using the STRING database^5^ and visualized using Cytoscape software. Receiver operating characteristic (ROC) curves and the area under the curve (AUC) were generated using the pROC R package. False positive rate (FPR) as the horizontal axis, true positive rate (TPR) as the vertical axis, CI represents the confidence interval. The expression levels of FDCSP and EGFR were visualized using the ggplot2 R package. Statistical significance was determined using the log-rank test and Wilcoxon test, with a *P*-value <0.05 considered statistically significant. Cancer Cell Line Encyclopedia (CCLE) was used to analyze gene expression in different cell lines.

### Somatic gene mutation landscape analysis

The R package maftools was used to analyze somatic mutation profiles in TNBC and non-TNBC patients. Somatic genes with mutation frequencies higher than 2 were screened. Genes with significantly higher mutation frequencies in each molecular subtype were then further identified using a Fisher’s exact test with a threshold of *P* < 0.05. Waterfall plots were then used to visualize the mutation status of the top 10 somatic genes in each molecular subtype.

### Immune cell infiltration analysis

To assess the proportions of immune cell populations within breast tissue samples, we uploaded formatted gene expression data to the CIBERSORT web portal. The analysis utilized the LM22 gene signature, a well-validated panel designed to sensitively and specifically distinguish 22 human hematopoietic cell phenotypes. CIBERSORT employs a deconvolution algorithm based on linear support vector regression, generating a *P*-value for each sample to evaluate the confidence of the deconvolution results. A *P*-value <0.05 was considered statistically significant and indicative of reliable quantification. The proportions of different immune cell populations were visualized using stacked bar charts.

### Single-cell RNA-seq data processing and quality control

The scRNA-seq data from the GSE161529 dataset were reanalyzed. Data processing was performed using the Read10X function from the R package Seurat (version 4.1.0). After merging data from all samples, cells with fewer than 400 or more than 4,000 expressed genes, as well as those with mitochondrial gene expression exceeding 5%, were excluded. Following filtration, the global scale normalization method LogNormalize was applied to ensure equal total gene expression levels across cells, with a scale factor set to 10,000. The FindVariableFeatures function was then used to identify the top 2,000 variable genes for downstream analysis. To mitigate batch effects between samples, the ScaleData, RunPCA, and Harmony functions were applied sequentially. Cell clustering was performed using FindNeighbors (dimensions 1–20) and FindClusters (resolution = 1.0). Unsupervised cluster analysis and visualization were conducted using uniform manifold approximation and projection (UMAP). Cell clusters were annotated based on known cell type marker genes using the FindAllMarkers function, with parameters set as follows: min.pct = 0.1, logfc.threshold = 0.25. Statistical significance was determined using the non-parametric Wilcoxon rank sum test with Bonferroni correction. The proportions of different cell clusters were visualized using stacked bar graphs generated with the ggplot2 R package.

### Spatial transcriptomic analysis

Data processing and visualization were performed using the R package Seurat. Specifically, we applied SCTransform for data normalization, RunPCA for dimensionality reduction, FindNeighbors and FindClusters for clustering ST spots, and RunUMAP for data visualization. The spatial distribution of gene expression levels was visualized using SpatialDimPlot and SpatialFeaturePlot. To integrate scRNA-seq data with ST data, we used FindTransferAnchors to identify anchor points between the datasets and TransferData to transfer cell type annotations from scRNA-seq to ST data.

### Cell–cell interaction analysis

CellPhoneDB, a publicly available repository of ligands, receptors, and their interactions, was used to analyze cell-cell communication. To quantify interaction frequencies between cell subsets, we employed the pheatmap function within the pheatmap R package. This analysis was conducted using the raw count matrix extracted from the Seurat object and a corresponding cell type annotation file. Additionally, the ktplots R package was utilized to predict and visualize the potential interaction strength between ligand-receptor pairs based on their average expression levels. Only statistically significant ligand-receptor pairs (*P-*values <0.01) were included for visualization.

The correlation between FDCSP and EGFR was analyzed using Gene Expression Profiling Interactive Analysis (GEPIA).^6^ Spearman correlation analysis was performed to calculate the *P-value* for the comparison.

### Predicting the immunotherapy response in the FDCSP subgroup

We employed the tumor immune dysfunction and exclusion (TIDE) method to evaluate the response probability of individuals to immunotherapy in TCGA-BRCA. The Wilcoxon test was used to compare the differences of TIDE-related scores among different FDCSP subgroups (*P*-values <0.05), and Chi-square test was used to compare the differences of therapeutic outcomes among different FDCSP subgroups.

### Computational analysis of resistance (CARE)

CARE^7^ [16] is a computational tool designed for large-scale extrapolation of response biomarkers and drug combinations for targeted therapies, utilizing compound screening data. A positive CARE score indicates higher gene expression associated with drug sensitivity, while a negative CARE score suggests drug resistance. We analyzed drugs targeting FDCSP using three databases: Cancer Cell Line Encyclopedia (CCLE), the Cancer Therapeutics Response Portal (CTRP), and the Cancer Genome Project (CGP). Drugs with a positive CARE score and a *P*-value <0.05 were identified as potential therapeutic candidates.

### Estimation of candidate drug for high-FDCSP patients

To further analyze the interactions between the identified drugs and FDCSP, we first obtained the structures of Quizartinib, from the PubChem database. The protein structures of the FDCSP was obtained from the AlphaFold and then docked using AutoDock. The higher scoring docking conformation was retained. If the molecular docking energy is less than −1.2 kcal/mol, we think the docking result is feasible.

### Immunohistochemistry (IHC) assay

Human breast tumor specimens and normal breast tissues were fixed with 4% paraformaldehyde for more than 48 h and paraffin embedded. The tissues were sectioned to 4 mm thickness for hematoxylin and eosin (H&E) and IHC. Tissue sections were first deparaffinized, hydrated in xylene and different concentrations of ethanol, and then placed in 3% hydrogen peroxide methanol to block endogenous peroxidase. Subsequently, tissues were antigenically repaired with citrate buffer (0.01 M, pH 6.0) and blocked with 10% (v/v) normal target serum for 30 min at room temperature. Tissues were incubated with FDCSP antibody (Solarbio, K107164P) overnight at 4 °C in a humidified environment, followed by incubation of the secondary antibody for 30 min at room temperature. Finally, tissues were incubated with 3,3-diaminobenzidine restained with hematoxylin, and then dehydrated in different concentrations of alcohol. Finally, the sections were covered with cover slips and observed under light microscope. FDCSP antibody were diluted by 1:400.

### Immunofluorescence (IF) assay

Tissue sections were deparaffinized, hydrated, and then permeabilized in 0.5% Triton X-100 for 10 min. The sections were blocked with 10% normal goat serum for 30 min, and then incubated with primary antibody in a humid chamber at 4 °C overnight. Using the TSA Fluorescent Triple Staining Kit (AFIHC024), HRP secondary antibody corresponding to the species of the primary antibody was added for 50 min under room temperature and light protection, TYR-520 fluorescent dye was added for 15 min, and washed three times with PBS. The above steps were repeated with 10% normal goat serum, and another primary and secondary antibody were added. TYR-570 fluorescent dye was added for 15 min and washed 3 times with PBS.DNA was restained with 4,6-diamidino-2-phenylindole (DAPI) in PBS for 10 min. Fluorescence was observed using an OLYMPUS, IX83-FV3000-OSR confocal microscope.

### Code availability

No algorithm or software was generated for this study. The code for reproducing major figure is available on GitHub.^8^ Any additional information required to reanalyze the data reported in this article is available from the lead contact upon request.

## Results

### Identification of differentially expressed genes related to TNBC

**FIGURE 1:**
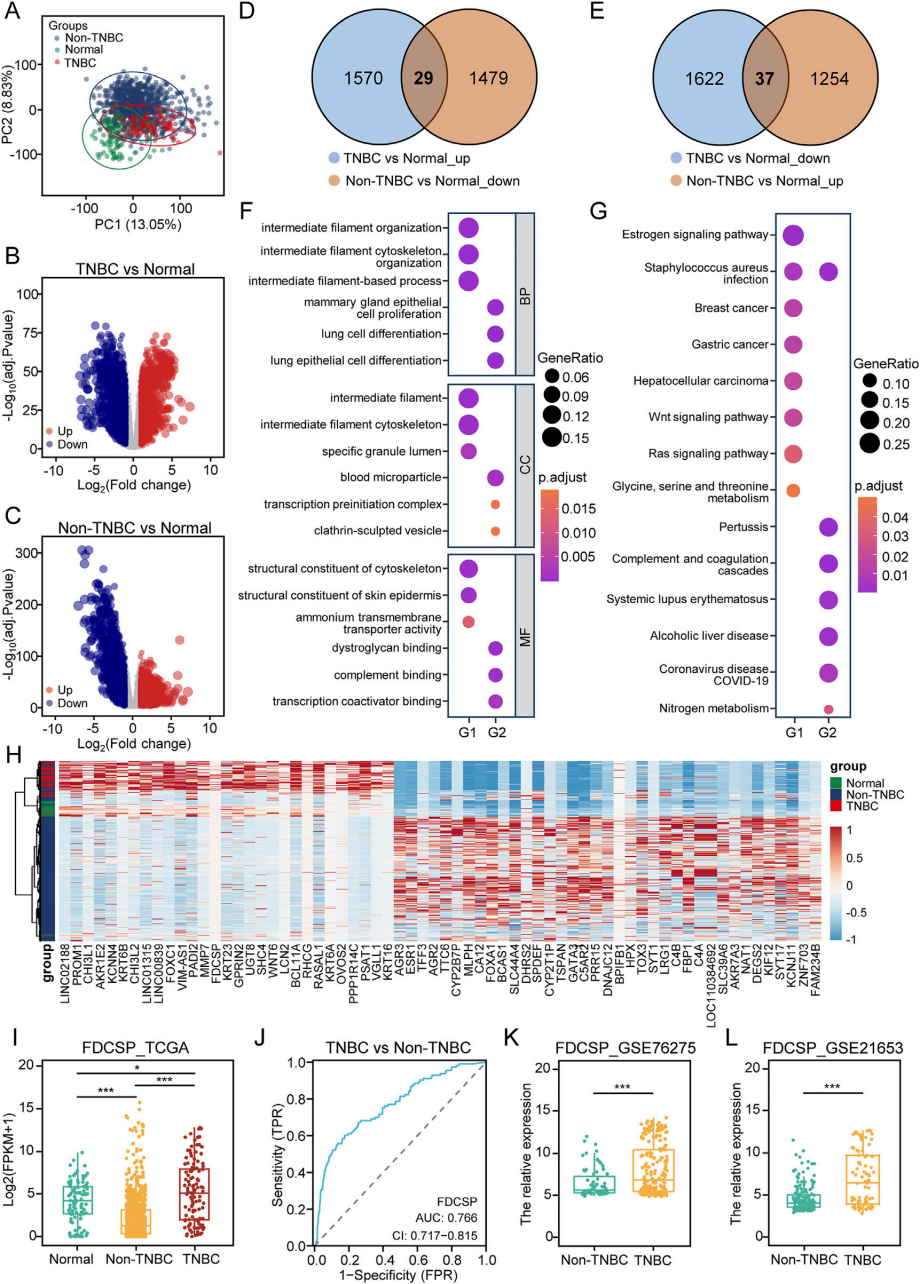
Differential analysis of TNBC and non-TNBC based on TCGA Data. **(A)** PCA of TNBC, non-TNBC and normal breast tissue samples from TCGA-BRCA cohort. **(B)** Volcano plot illustrating DEGs identified in TCGA data by comparing TNBC to normal tissue. (Blue: down-regulated DEGs; Red: up-regulated DEGs; Grey: unchanged genes; Adjusted *P*-value <0.05 and |log2FC| > 1). **(C)** Volcano plot illustrating DEGs identified by comparing non-TNBC to normal tissue. **(D)** Venn diagram showing the overlap of DEGs up-regulated in TNBC versus normal tissue, but down-regulated in non-TNBC versus normal tissue. **(E)** Venn diagram showing the overlap of DEGs down-regulated in TNBC versus normal tissue, but up-regulated in non-TNBC versus normal tissue. **(F)** GO functional enrichment analysis of gene lists G1 and G2. **(G)** KEGG pathway enrichment analysis of gene lists G1 and G2. **(H)** Heatmap showing the expression levels of DEGs in G1 and G2. **(I)** Expression of FDCSP in the TCGA-BRCA cohort. **(J)** ROC curve for FDCSP as a diagnostic marker. **(K)** Relative expression of FDCSP in GSE76275. **(L)** Relative expression of FDCSP in GSE21653. *P* values were determined using Wilcox tests in **(I)**, **(K)** and **(L)**. *P* value <0.05 was considered statistically significant (*P < 0.05, **P < 0.01, ***P < 0.001).

To identify genes differentially expressed in TNBC, we categorized the TCGA-BRCA dataset into TNBC, non-TNBC, and normal breast tissue groups for differential expression analysis. PCA revealed significant inter-group differences and minimal intra-group variability, confirming the suitability of the samples for comparative analysis (Figure 1A). Using the Limma package, we identified DEGs between TNBC and normal tissue, and between non-TNBC and normal tissue (adjusted *P*-value <0.05, |log2FC| > 1). Volcano plots visualized these results (Figures 1B,C). Compared to normal breast tissue, TNBC exhibited 1599 upregulated genes and 1659 downregulated genes, while non-TNBC showed 1291 upregulated genes and 1508 downregulated genes.

Venn diagram analysis identified 29 genes (G1) upregulated in TNBC but downregulated in non-TNBC (Figure 1D), and 37 genes (G2) downregulated in TNBC but upregulated in non-TNBC (Figure 1E). These two gene sets were selected for further investigation. GO analysis revealed that G1 genes were associated with intermediate filament organization and structural constituents of the cytoskeleton, while G2 genes were linked to mammary gland epithelial cell proliferation, complement binding, and transcription coactivator binding (Figure 1F). KEGG pathway enrichment analysis indicated that the DEGs were involved in multiple signaling pathways. Specifically, G1 genes were enriched in the estrogen, Wnt, and Ras signaling pathways, whereas G2 genes were enriched in pathways related to *Staphylococcus aureus* infection and complement and coagulation cascades (Figure 1G). A heatmap visualized the expression patterns of these 66 DEGs across the samples (Figure 1H). PPI networks for G1 and G2 were constructed to analyze their interactions using STRING analysis (Supplementary Figures S1A,B).

Of course, we also directly analyzed the differential genes between TNBC and non-TNBC groups in the TCGA-BRCA cohort and the GEO dataset, visualizing the results using volcano plots (Supplementary Figures S1C,D). In the TCGA-BRCA cohort, 3287 DEGs were identified, while the GSE76275 cohort yielded 315 DEGs. Venn diagram analysis revealed 108 co-upregulated DEGs and 148 co-downregulated DEGs shared between the two datasets (Supplementary Figures S1E,F). GO analysis demonstrated that the co-upregulated genes were primarily involved in epidermis development and intermediate filament organization. Conversely, the co-downregulated genes were associated with negative regulation of platelet-derived growth factor receptor (PDGFR) signaling pathway and monooxygenase activity (Supplementary Figure S1G). KEGG analysis indicated that the co-upregulated DEGs were enriched in the estrogen and Wnt signaling pathways, while the co-downregulated DEGs were linked to the peroxisome proliferator-activated receptor (PPAR) signaling pathway and cytochrome P450 metabolism (Supplementary Figure S1H).

Based on these two screening methods, we identified FDCSP as a gene common to both the G1 gene set and the co-upregulated DEGs. Consequently, we selected FDCSP for further investigation. Analysis of the TCGA-BRCA dataset revealed that FDCSP expression was significantly upregulated in TNBC compared to normal tissues, while it was downregulated in non-TNBC, with a notable difference between the two groups (Figure 1I). ROC curve analysis demonstrated that FDCSP could effectively distinguish TNBC from non-TNBC, with an AUC of 0.766 (Figure 1J). To validate these findings, we further analyzed FDCSP expression in the GSE76275 and GSE21653 datasets, which yielded consistent results (Figures 1K,L).

### The role of FDCSP in TNBC

Using TCGA data, we examined the relationship between FDCSP expression levels and TNM staging in breast cancer. Our analysis revealed that FDCSP expression showed statistically significant differences (*P* < 0.05) only between T2 and T4 stages in non-TNBC cases. Notably, no significant variations in FDCSP expression were observed across different TNM stages in TNBC patients (Supplementary Figures S2A–F).

**FIGURE 2:**
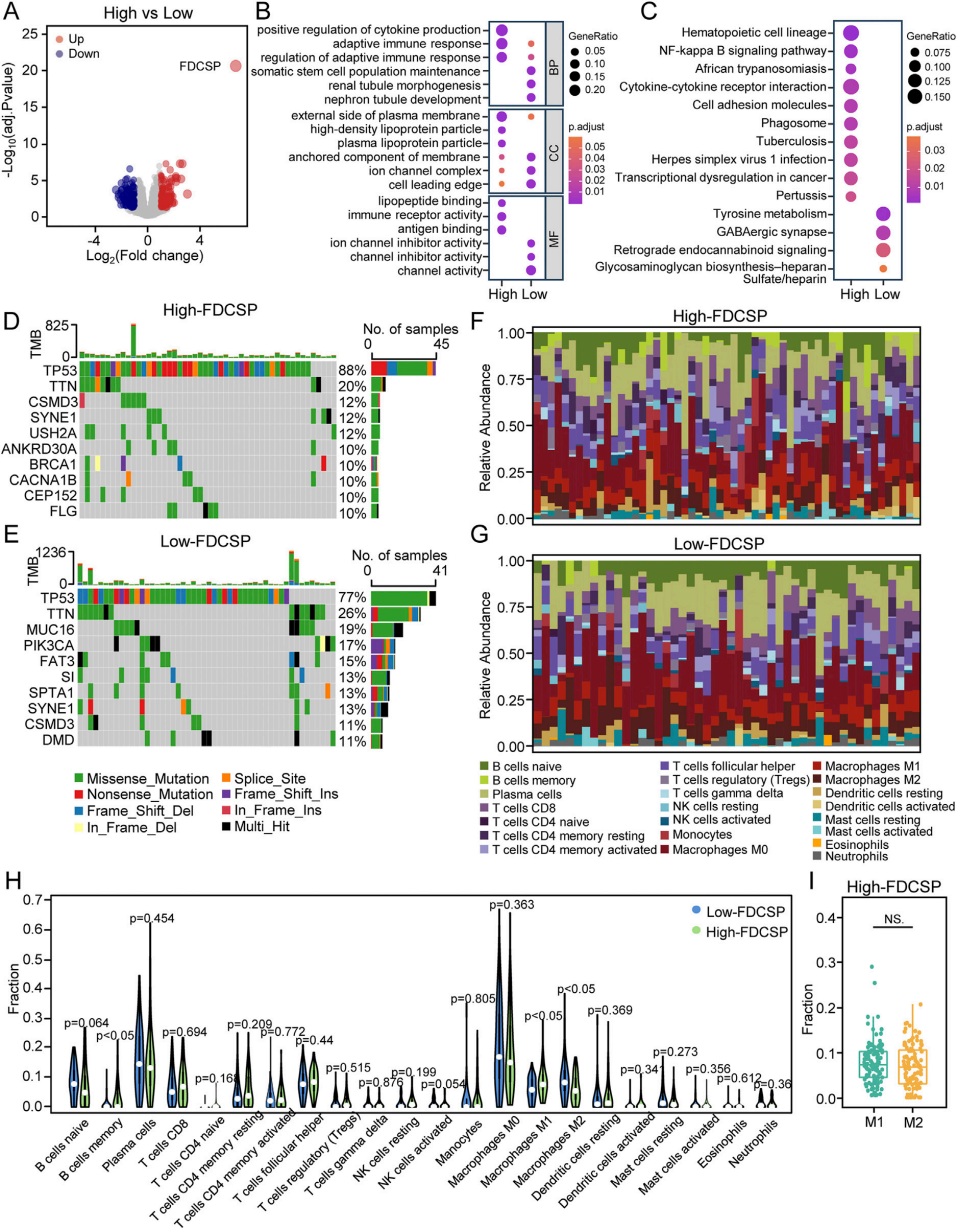
Functional differences between high and low FDCSP expression groups in TNBC. **(A)** Volcano plot illustrating DEGs in TNBC identified by comparing the high-FDCSP expression group to the low-FDCSP expression group. **(B)** GO analysis of DEGs from the high-FDCSP group and the low-FDCSP group. **(C)** KEGG analysis of DEGs. **(D)** Oncoplots showing mutated genes in the high-FDCSP expression group. **(E)** Oncoplots showing mutated genes in the low-FDCSP expression group. **(F)** Proportions of immune cells in high-FDCSP expression TNBC samples. **(G)** Proportions of immune cells in low-FDCSP expression TNBC samples. **(H)** Violin plot illustrating differences in immune cell infiltration between the high-FDCSP expression group and the low-FDCSP expression group. **(I)** Box plot illustrating differences between M1 and M2 macrophage infiltration in TNBC. *P*-values were determined using Wilcoxon tests. NS.: no significance.

To elucidate the role of FDCSP in TNBC, we stratified TNBC samples in TCGA based on FDCSP expression levels. Samples were divided into high and low FDCSP expression groups according to the median expression of the FDCSP gene. Differential gene expression analysis between these two groups was then performed (Figure 2A). GO analysis of DEGs in the high-FDCSP group revealed associations with biological processes, including positive regulation of cytokine production and adaptive immune response (Figure 2B). KEGG analysis indicated that DEGs in the high-FDCSP group were enriched in pathways such as the NF-κB signaling pathway, cytokine-cytokine receptor interaction, cell adhesion molecules, and transcriptional dysregulation in cancer (Figure 2C).

Then, we further investigated the top ten genes with the highest mutation rates in both the high and low FDCSP expression groups in TCGA. TP53, the most frequently mutated gene in breast cancer, showed a markedly higher mutation rate (88%) in the high-FDCSP group compared to the low-FDCSP group (Chi-square test: *P* < 0.05). In addition to TP53, ANKRD30A, BRCA1, and CACNA1B were among the top ten mutated genes in the high-FDCSP group, whereas MUC16 and PIK3CA were prominent in the low- FDCSP group (Figures 2D,E). These results suggest a potential link between FDCSP expression and somatic mutations in TNBC.

Considering the close association of FDCSP with the immune system, we examined the relationship between FDCSP expression and immune cell infiltration in TNBC. Immune infiltration profiles were generated for both the high and low FDCSP expression groups (Figures 2F,G), and differences in immune cell composition were compared (Figure 2H). Notably, high FDCSP expression was associated with increased memory B cell, M1 macrophage infiltration, and decreased M2 macrophage infiltration (Figure 2H). However, when we compared the infiltration between M1 macrophage and M2 macrophage in high FDCSP expression groups, we found that there was no difference between the two. This indicates that FDCSP high expression promotes M1 macrophage infiltration, but M2 macrophages are still present in tumors with FDCSP high expression (Figure 2I).

### A high-FDCSP basal subset is identified in TNBC

**FIGURE 3:**
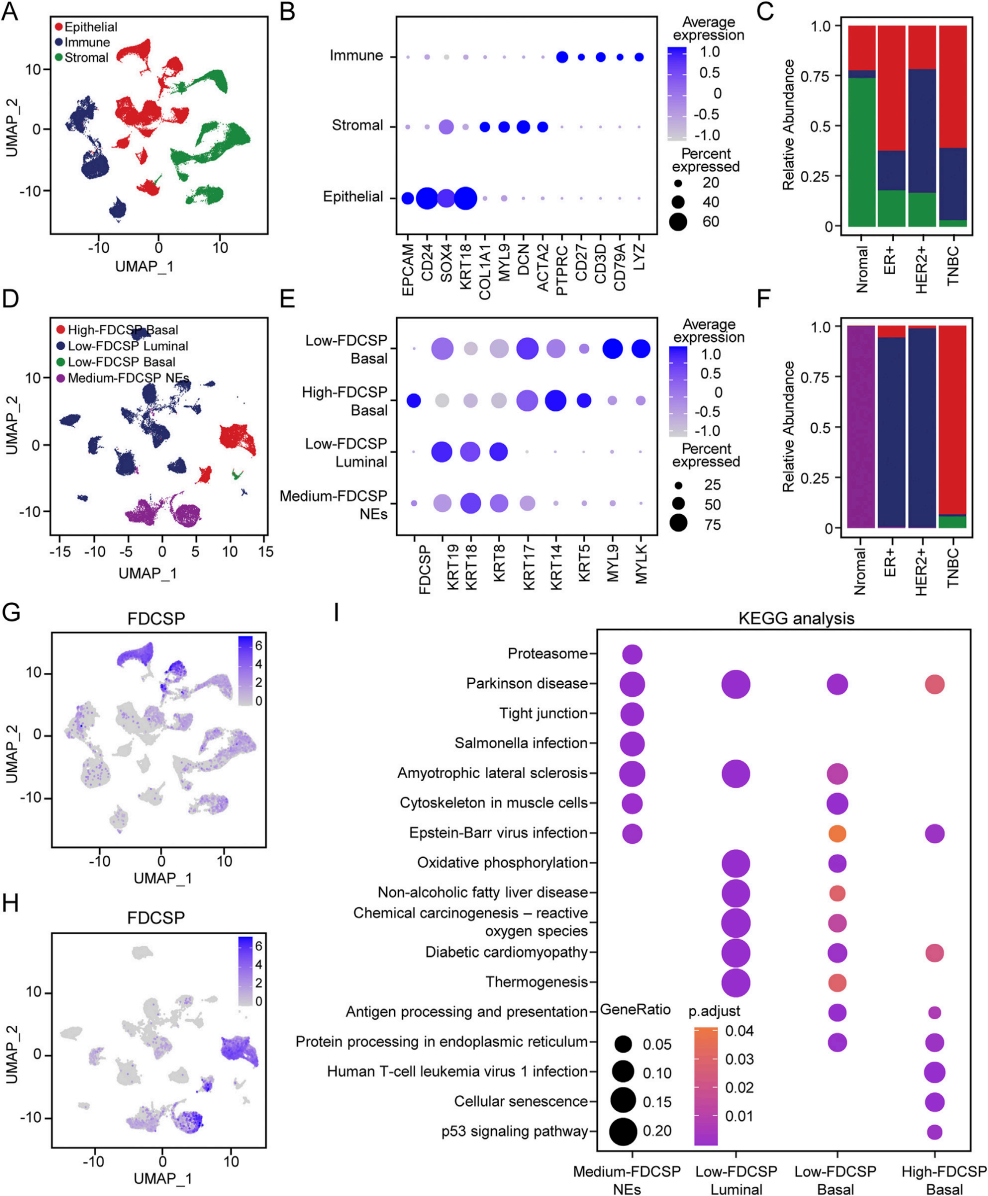
Expression pattern of FDCSP at single cell level in breast cancer. **(A)** UMAP representations of all scRNA-seq data from GSE161529, include including samples from normal breast tissue, ER+, HER2+ breast cancer and TNBC. **(B)** Dot plot showing the average expression of known markers in indicated clusters. **(C)** Bar plot showing the percentage of each cell subtypes. Colors correspond to those used in panel **(A)**. **(D)** UMAP representation showing the composition of epithelial subtypes. **(E)** Dot plot showing the expression of marker genes for each epithelial subtype. **(F)** Bar plot showing the percentage of each epithelial subtype. Colors correspond to those used in panel **(E)**. **(G)** Feature plot showing FDCSP expression in all scRNA-seq data from GSE161529. **(H)** Feature plot showing FDCSP expression in epithelial subtypes. **(I)** KEGG analysis of different epithelial subtypes.

The TME is critical in the initiation, progression, invasion, and metastasis of TNBC, significantly impacting patient prognosis. To investigate FDCSP expression within the breast cancer microenvironment, we analyzed the scRNA-seq dataset GSE161529, which comprises normal breast tissue, ER+ breast cancer, HER2+ breast cancer, and TNBC samples. All cell populations were categorized into three primary types based on established genetic markers: epithelial cells (high EPCAM, CD24, SOX4, and KRT18), stromal cells (high COL1A1, MLY9, DCN, and ACTA2), and immune cells (PTPRC (CD45), CD27, CD3D, CD79A, and LYZ) (Figures 3A,B). The proportions of these cell types varied across the four sample groups and fibroblasts are least prevalent in TNBC (Figure 3C; Supplementary Figure S3A).

FDCSP was expressed in all three cell types, with the highest levels observed in epithelial cells (Figure 3G). In normal breast tissue, FDCSP expression was primarily detected in epithelial and stromal cells. In contrast, in TNBC, FDCSP expression was predominantly localized to epithelial cells (Supplementary Figure S3B). Therefore, we further clustered and annotated epithelial cells based on FDCSP expression levels. Epithelial cells predominantly from normal tissue were classified as normal epithelial cells (NEs) based on their distinct distribution patterns (Figure 3F). The remaining epithelial cell clusters were categorized as luminal (high KRT19, KRT18 and KRT8) or basal cells (high KRT17, KRT14, KRT5, MYL9 and MYLK) using established markers (Figures 3D,E). FDCSP exhibited moderate expression in NEs. Luminal cells showed low FDCSP expression, whereas basal cells, which were the predominant epithelial cell type in TNBC, displayed elevated FDCSP expression in most cases (Figure 3F; Supplementary Figure S3C). Figure 3H illustrated the distribution of FDCSP expression across epithelial cell subtypes. Given the diverse functional roles of different epithelial cells within the TME, KEGG analysis was performed based on the DEGs of different epithelial cell mentioned above to elucidate their potential contributions (Figure 3I). Notably, basal cells with high FDCSP expression were functionally enriched in pathways related to cellular senescence and the p53 signaling pathway. This finding is consistent with our previous observation that breast cancer patients with high FDCSP expression exhibited an increased TP53 mutation rate, suggesting a potential link between FDCSP and dysregulation of the p53 signaling pathway.

### The validation of high-FDCSP basal subpopulation

**FIGURE 4:**
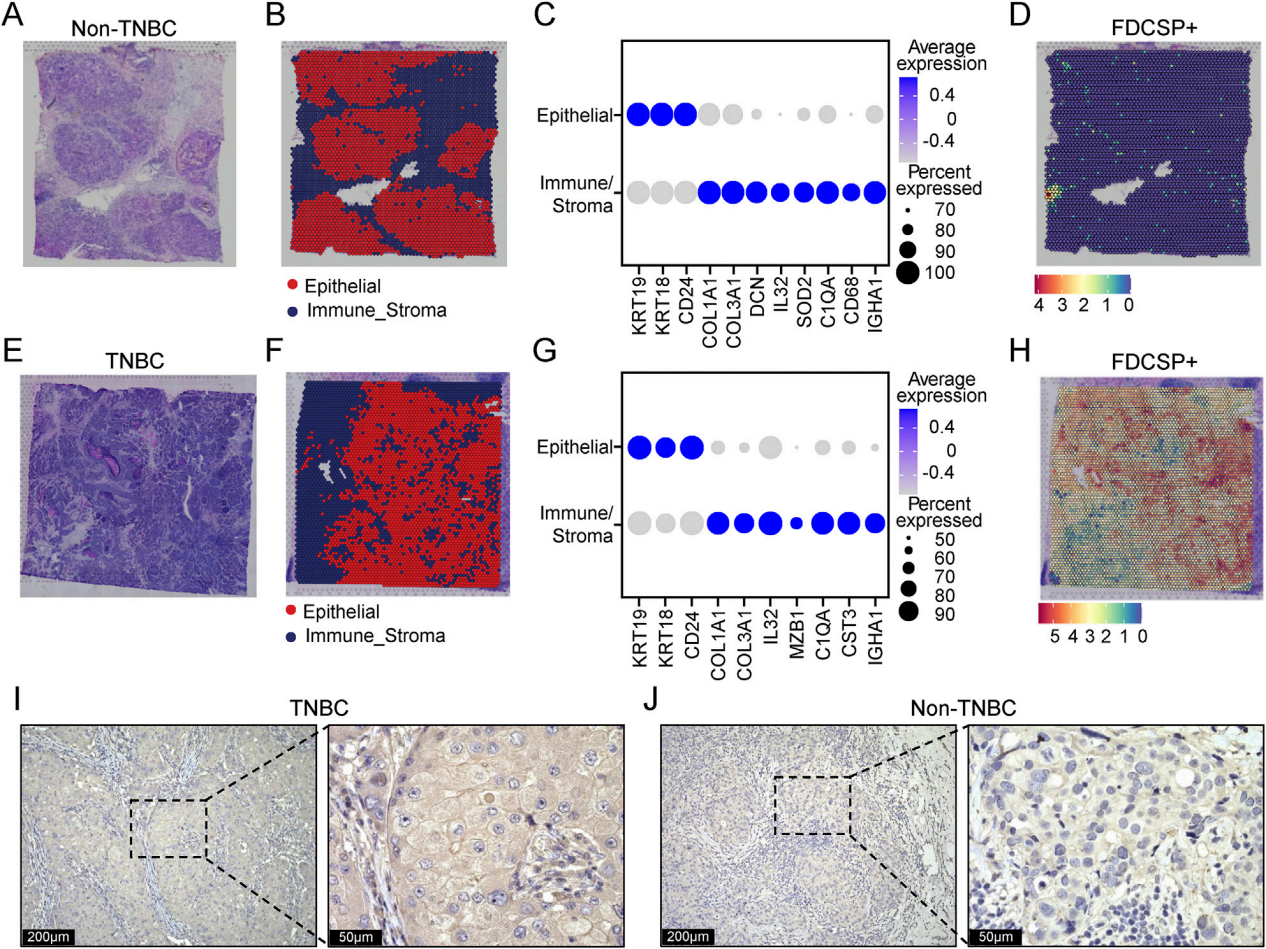
Spatial expression patterns of FDCSP in TNBC and non-TNBC. **(A)** H&E stained image of non-TNBC tissue. **(B)** Unbiased clustering of ST spots, identifying epithelial cells and immune-stroma cells in non-TNBC tissue. **(C)** Dot plot showing the expression of marker genes in epithelial cells and immune-stroma cells. **(D)** Spatial distribution of FDCSP gene expression in non-TNBC tissue. **(E)** H&E stained image of TNBC tissue. **(F)** Unbiased clustering of ST spots, identifying epithelial cells and immune-stroma cells in TNBC tissue. **(G)** Dot plot showing the expression of marker genes in epithelial cells and immune-stroma cells. **(H)** Spatial distribution of FDCSP gene expression in TNBC tissue. **(I)** IHC staining showing the expression of FDCSP in TNBC tissues. **(J)** IHC staining showing the expression of FDCSP in non-TNBC tissues.

To further validate FDCSP expression in BRCA, we analyzed the expression of cell lines from CCLE databases, which showed a low expression of FDCSP in non-TNBC cell lines, while a high expression in TNBC cell lines (Supplementary Table S1). ST enables the visualization and quantitative analysis of the transcriptome with spatial resolution within tissue sections, overcoming the limitations of scRNA-seq, which lacks spatial information. We obtained ST data for TNBC and non-TNBC samples from the 10x Genomics website and previous studies [15]. Based on H&E staining (Figures 4A,E), unbiased clustering, and marker gene expression (Figures 4B,F), we categorized the tumor tissue into two distinct regions: the epithelial region, characterized by high expression of KRT19, KRT18, and CD24, and the immune-stromal region, marked by high expression of COL1A1, COL3A1, IL32, C1QA, and other related genes (Figures 4C,G). In TNBC, regions with high FDCSP expression predominantly overlapped with the distribution of epithelial cells (Figure 4H). In contrast, FDCSP expression was rarely observed in non-TNBC samples (Figure 4D). Subsequently, we evaluated the expression level of FDCSP in clinical TNBC and non-TNBC tissue samples using IHC staining. The results demonstrated that the staining intensity of FDCSP in TNBC tissues was significantly greater than that observed in non-TNBC tissues (Figures 4I,J; Supplementary Figure S2G). These findings are consistent with our previous scRNA-seq results, further validating the specific association of FDCSP with TNBC epithelial cells.

### Cell-cell interactions in the breast cancer microenvironment

**FIGURE 5:**
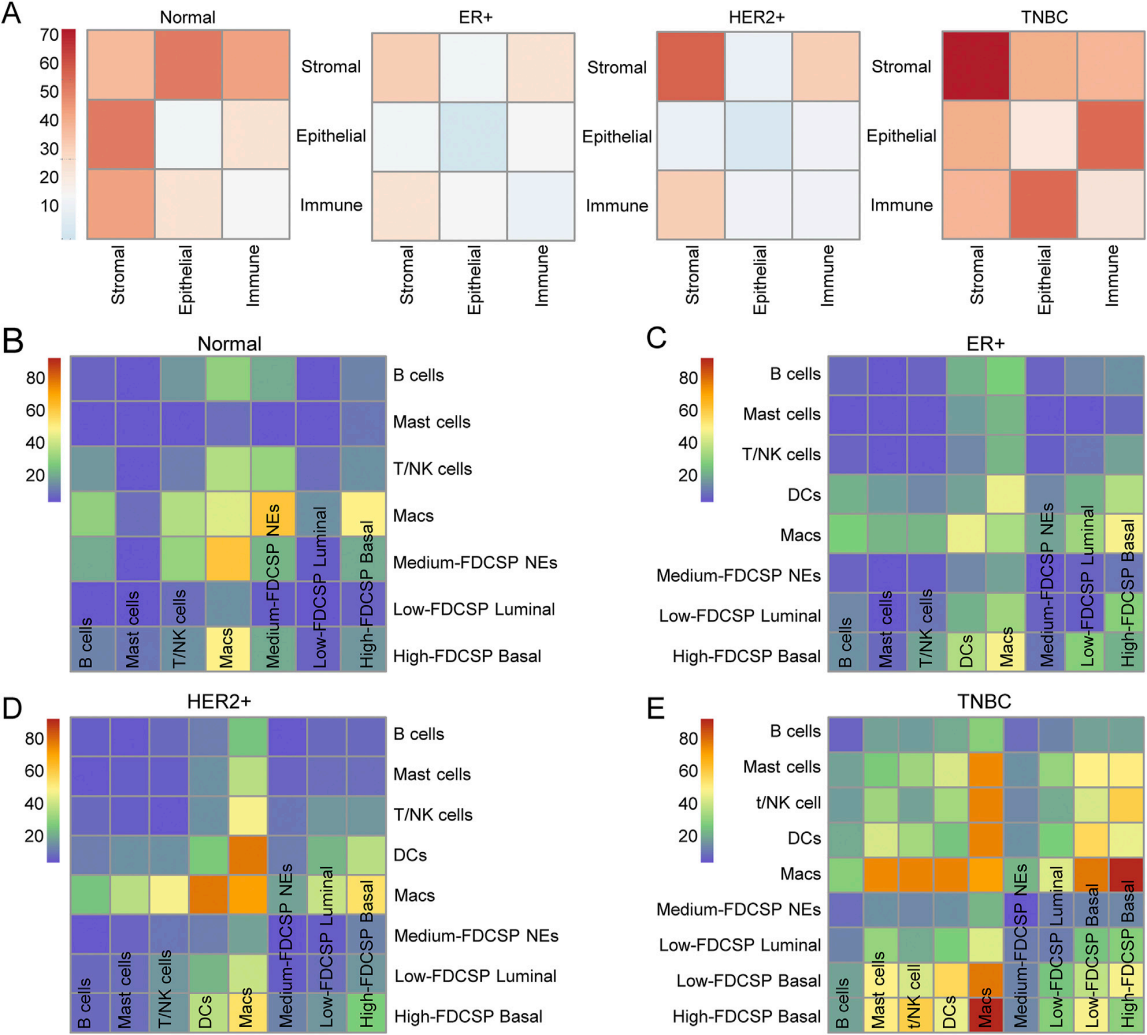
Interaction between epithelial and immune cells. **(A)** The mutual interactions among the main TME components in normal, ER+, HER2+ and TNBC samples. **(B)** Heatmap showing the number of cell-cell interactions between immune subtypes and epithelial subtypes in normal samples, as predicted by CellphoneDB. **(C)** Heatmap showing the number of cell-cell interactions between immune subtypes and epithelial subtypes in ER+ samples, as predicted by CellphoneDB. **(D)** Heatmap showing the number of cell-cell interactions between immune subtypes and epithelial subtypes in HER2+ samples, as predicted by CellphoneDB. **(E)** Heatmap showing the number of cell-cell interactions between immune subtypes and epithelial subtypes in TNBC samples, as predicted by CellphoneDB.

Given the critical role of the TME in tumor progression and therapeutic response, we conducted a CellPhoneDB-based cell interaction analysis to evaluate interactions between epithelial cells and other cell types. The analysis revealed that interactions between epithelial cells and immune cells were predominant in the TME (Figure 5A). To further characterize these interactions, we performed dimensionality reduction, clustering, and annotation of immune cells. Based on marker gene expression, immune cells were classified into B cells, dendritic cells (DCs), T/natural killer (NK) cells, mast cells, and macrophages (Macs) (Supplementary Figures S4A–C).

We then examined the interactions between these immune cell types and different epithelial cell populations (Figures 5B–E). The results demonstrated that, in breast cancer tissues, macrophages exhibited the highest number of interactions with other cell types. Notably, in TNBC, the interaction between high-FDCSP basal cells and macrophages was the most frequent, suggesting that these two cell types play a central role in cellular communication within the TNBC microenvironment.

### Macrophages interact with high-FDCSP basal cells via TGFβ1-EGFR

**FIGURE 6:**
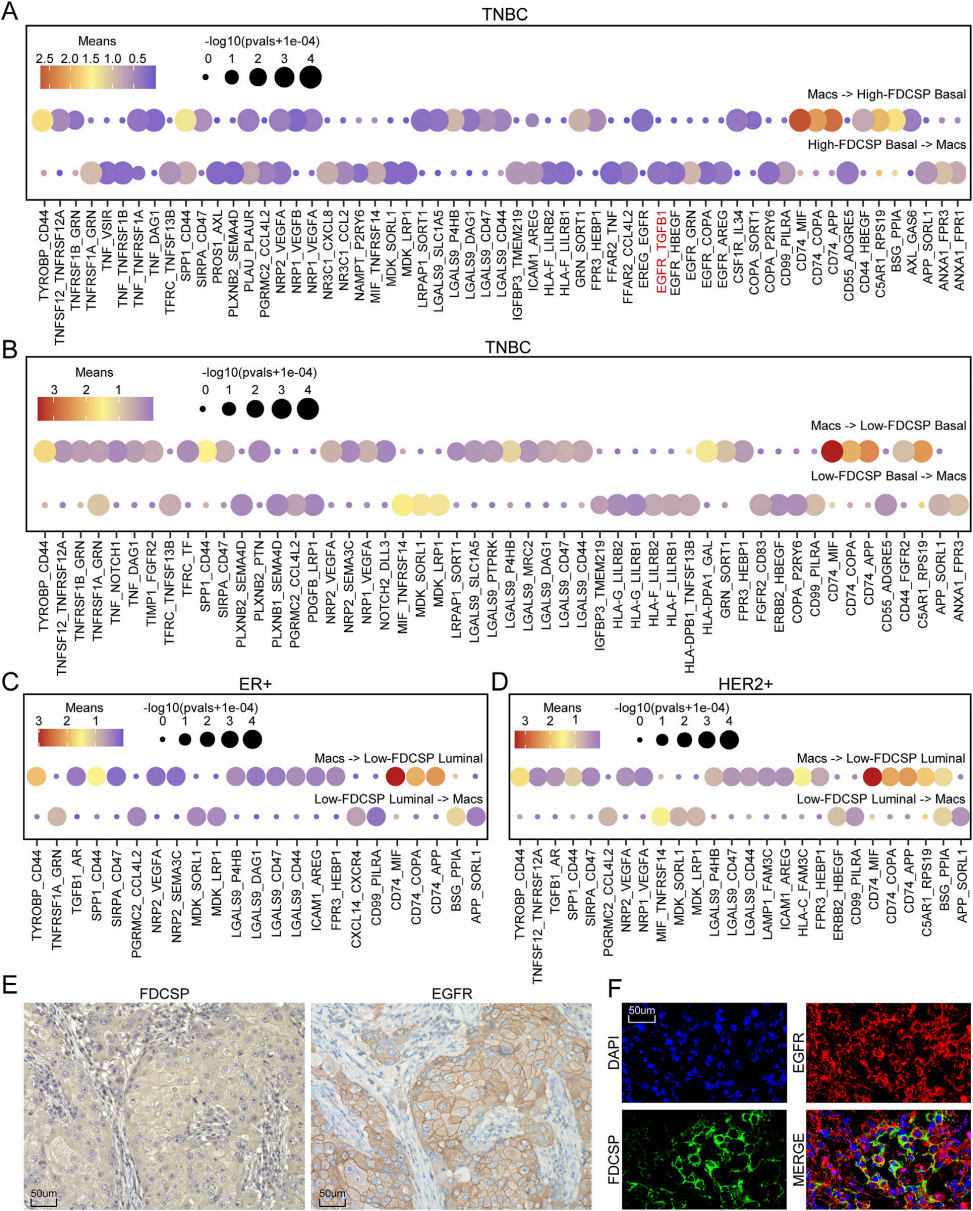
Interaction between FDCSP-positive epithelial cells and macrophages. **(A)** Ligand-receptor pairs involved in mutual interactions between macrophages and basal cells with high FDCSP expression in TNBC. **(B)** Ligand-receptor pairs involved in mutual interactions between macrophages and basal cells with low FDCSP expression in TNBC. **(C)** Ligand-receptor pairs involved in mutual interactions between macrophages and luminal cells with low FDCSP expression in ER+ breast cancer. **(D)** Ligand-receptor pairs involved in mutual interactions between macrophages and luminal cells with low FDCSP expression in HER2+ breast cancer. **(E)** IHC staining showing the co-expression of EGFR and FDCSP in TNBC tissues. **(F)** IF assay showing the co-expression of EGFR and FDCSP within the same TNBC tissue sections.

Considering that it is not possible to further subdivide macrophages into M1 and M2 types (Supplementary Figures S4D–H; Supplementary Table S2), we used all macrophage data for our study. Further analyses were conducted to characterize the interactions between macrophages and high-FDCSP/low-FDCSP basal cells in TNBC and between macrophages and low-FDCSP luminal cells in ER+ and HER2+ breast cancer. The results revealed that, in TNBC, macrophages could interact with EGFR on high-FDCSP basal cells by secreting TGF-β1 (Figure 6A). This specific interaction was not observed in low-FDCSP basal cells or low-FDCSP luminal cells from ER+ and HER2+ breast cancer samples (Figures 6B–D). TGF-β is known to transactivate EGFR and promote breast cancer migration and invasion through the Smad3 and ERK/Sp1 signaling pathways. Therefore, we sought to explore the potential connection between FDCSP and EGFR. EGFR expression was significantly higher in TNBC compared to non-TNBC (Supplementary Figure S5A) and was detected in both NEs and basal cells of TNBC (Supplementary Figure S5B). Co-localization analysis demonstrated that EGFR and FDCSP were co-expressed in epithelial cells (Supplementary Figure S5C), and their expression levels were positively correlated (Supplementary Figure S5D). This finding was further validated at the tissue level of TNBC through IF and IHC staining (Figures 6E,F).

### The role of FDCSP in immunotherapy response and target drug prediction

**FIGURE 7:**
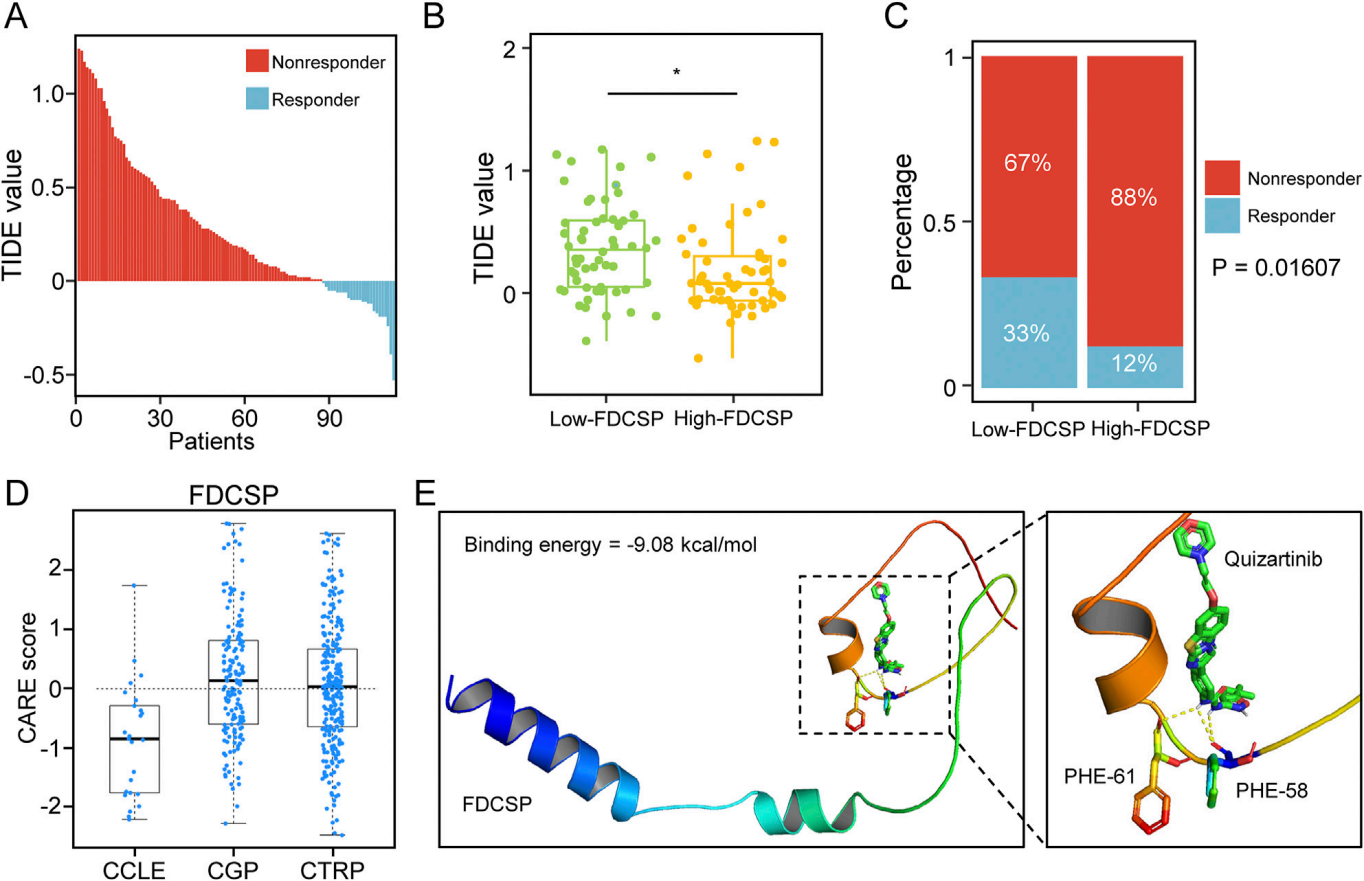
Predictive value of FDCSP in breast cancer immunotherapy and targeted drug response. **(A)** TIDE scores in TCGA TNBC patients. **(B)** TIDE scores in different FDCSP subgroups. *P*-values were determined using Wilcoxon tests. *: *P* < 0.05. **(C)** The effect of ICB treatment in different FDCSP subgroups. *P*-values were determined using Chi-square test. **(D)** CARE analysis of FDCSP in the CCLE, CTRP, and CGP databases. **(E)** 3D docking model of FDCSP and Quizartinib compound molecule prediction.

We employed TIDE to estimate immunotherapy efficacy in high and low FDCSP subgroups (Figure 7A). Lower TIDE scores, suggesting a reduced likelihood of immune evasion, were observed in the low-FDCSP group, indicating a potentially greater benefit from immunotherapy in this subgroup (Figure 7B). Chi-square tests also found that the low-FDCSP group benefited more from ICB treatment (Figure 7C). This suggests that FDCSP could serve as a valuable biomarker for identifying patients who may benefit from ICB treatment. Additionally, we utilized the CARE database to explore the relationship between FDCSP expression and drug efficacy (Figure 7D; Supplementary Table S3). Among the drugs analyzed, six from the CGP and eight from the CCLE were evaluated. Positive CARE scores were observed for four and five drugs, respectively, indicating that these drugs are likely to be more effective in patients with high FDCSP expression. To further analyze the drugs identified, we performed molecular docking of the FDCSP with the most likely effective drug. The docking models of FDCSP with Quizartinib are shown in Figure 7E, where the binding energy = −9.08 kj/mol. Existing study have found that therapy-induced senescence TNBC cells (MDA-MB-231, Hs578T) remained sensitive to Quizartinib [17]. Our molecular docking results indicate that the binding energy is far less than −2.5 kj/mol, suggesting that Quizartinib can treat triple-negative breast cancer by targeting FDCSP specifically.

## Discussion

TNBC is a particularly aggressive subtype of breast cancer, associated with a poorer prognosis and higher mortality rate compared to non-TNBC. This subtype is defined by the absence of ER, PR, and HER2 expression, which limits the efficacy of endocrine therapies and HER2-targeted treatments [18]. Consequently, the identification of novel therapeutic targets and the development of effective treatment strategies for TNBC are critically important. In this study, we aimed to identify key genes that differentiate TNBC from non-TNBC. Analysis of the TCGA-BRCA cohort revealed 29 DEGs that were upregulated in TNBC and downregulated in non-TNBC, relative to normal mammary tissue. ROC curve analysis indicated that FDCSP exhibits strong potential for distinguishing TNBC from non-TNBC. This differential expression was subsequently validated in two independent datasets, GSE76275 and GSE21653. Based on these findings, we selected FDCSP for further investigation.

The human FDCSP gene, located on chromosome 4, encodes a secreted protein expressed in FDCs, periodontal ligaments, and conjunctival epithelium [19, 20]. FDCSP exhibits a unique expression pattern within the immune system and preferentially binds to activated B cells. It may play a role in autoimmune conditions by modulating B cell immune responses [21]. Previous studies have shown that FDCSP overexpression reduces the expression of osteogenic genes in human periodontal ligament cells (hPDLCs) while increasing the expression of osteoclast-related genes, thereby promoting osteoclastogenesis [22]. FDCSP also influences periodontal ligament (PDL) cell proliferation and acts as a phenotypic stabilizer of fibroblasts by inhibiting their differentiation into mineralized tissue-forming cells [23]. Transcription of the FDCSP gene is stimulated by pro-inflammatory cytokines, including TNF-α, IL-1β, and IL-6, which target the FDCSP gene promoter [24–26]. In patients with immunoglobulin A nephropathy (IgAN), FDCSP expression is significantly reduced in the tonsils and negatively correlated with increased IgA production [27]. FDCSP may regulate germinal center B cells, control IgA production in B cells [28], and participate in the modulation of IgA production in IgAN tonsils. Due to these immunomodulatory functions, FDCSP is considered a promising candidate for therapeutic targeting [29].

FDCSP is abnormally overexpressed in several malignant tumors, including HPV+ HNSC [12], epithelial ovarian cancer (EOC) [11], endometrial cancer, lung adenocarcinoma [14], and RCC [13]. In contrast, it is nearly absent in equivalent benign lesions or normal tissues. Studies have demonstrated that FDCSP promotes the invasion and metastasis of ovarian cancer cells [11]. *In vitro*, FDCSP enhances the migration and aggressiveness of EOC cells and reduces intercellular adhesion by phosphorylating Akt at S473 and downregulating E-cadherin. Additionally, silencing FDCSP has been shown to induce cytoskeletal reorganization. These findings position FDCSP as a promising candidate for anti-tumor targeting. However, the role of FDCSP in breast cancer, particularly TNBC, remains unexplored. Therefore, we employed bioinformatics approaches to investigate the biological functions and potential regulatory mechanisms of FDCSP in TNBC.

In our study, TNBC samples were stratified into high- and low-expression groups based on FDCSP expression levels for differential analysis. GO and KEGG pathway analyses revealed that DEGs in the high-FDCSP group were associated with adaptive immunity, the NF-κB signaling pathway, and the positive regulation of cytokine production. Somatic gene mutation landscape analysis indicated an increased TP53 mutation rate in the high-FDCSP group. Immune infiltration analysis demonstrated elevated infiltration of B cells and M1 macrophages in the high-FDCSP group. Furthermore, single-cell transcriptomic analysis was performed to annotate and cluster breast cancer cells. The results showed that FDCSP was predominantly expressed in basal cells of TNBC tumors and exhibiting high expression, while it was rarely detected in non-TNBC epithelial cells. ST data analysis confirmed that FDCSP+ cells were highly expressed within the epithelial cell distribution range in TNBC but were scarce in non-TNBC, aligning with previous findings.

The TME is a complex ecosystem comprising multiple interacting cell populations. Previous studies have emphasized the critical role of the TME in key cancer-related processes, including tumor progression, treatment resistance, angiogenesis, and metastasis [30–32]. Mechanistically, the TME influences cancer cells through dynamic and intricate pathways that regulate cancer-associated signaling [33], such as ligand-receptor interactions, cytokine/metabolite signaling, and extracellular matrix (ECM) remodeling [34–38]. To further elucidate the TME in TNBC, we employed CellPhoneDB to quantitatively analyze cell-cell interactions. Our analysis revealed that interactions between epithelial cells and immune cells were the most prominent in TNBC. Further investigation demonstrated that basal cells exhibiting high FDCSP expression displayed the most significant communication with macrophages, strongly suggesting a close interaction between these cell types.

Macrophages, key cellular components of the TME, influence cancer progression and outcomes in diverse ways owing to their phenotypic plasticity [39, 40]. Tumor-associated macrophages (TAMs) can promote inflammation and exert anti-tumor effects. Conversely, they can also support tumor progression by facilitating angiogenesis [41, 42], promoting metastasis [43, 44], and suppressing T cell function [45, 46]. In breast cancer, TAM infiltration is associated with a poorer prognosis [47]. Our findings regarding the extensive crosstalk between high-FDCSP basal cells and macrophages offer potential novel insights into the mechanisms underlying TNBC malignancy.

Additionally, ligand-receptor pair analysis indicated that macrophages secrete TGF-β1, which interacts with the highly expressed EGFR on high-FDCSP basal cells. TGF-β signaling exhibits a dual role in cancer, demonstrating both tumor-suppressive and tumor-promoting effects depending on the specific context [48]. In early-stage breast cancer, TGF-β can act as a potent inhibitor of proliferation and inducer of apoptosis [49]. However, in advanced stages, it often promotes cancer aggressiveness. Many of these functions are mediated through the Smad signaling pathway [50–52]. The EGF/EGFR signaling pathway is a well-established driver of tumorigenesis [53]. Dysregulation of EGFR signaling has been observed in various cancers, including breast cancer [54], colon cancer [55], and lung cancer [56]. Overexpression of EGFR is not only associated with cancer progression but also correlates with a poorer prognosis in cancer patients [57, 58]. The interplay between EGF and TGF-β signaling exemplifies oncogenic cooperation and context-dependent regulation. In breast cancer, TGF-β expression is positively correlated with EGFR expression. TGF-β transactivates EGFR and promotes breast cancer migration and invasion through the Smad3 and ERK/Sp1 signaling pathways [59]. The downstream signaling pathways of EGFR play a crucial role in regulating cell cycle progression and the survival of mammary epithelial cells.

TNBC exhibits a significant propensity for metastasis, and patients who do not respond to chemotherapy typically experience a poor prognosis [60]. Immunotherapy, including the use of immune checkpoint inhibitors (ICIs) targeting molecules such as cytotoxic T-lymphocyte-associated antigen 4 (CTLA-4), programmed cell death protein 1 (PD-1), its ligand (PD-L1), and lymphocyte activation gene 3 (LAG-3), has shown improved efficacy and precision in targeting cancer cells [61–63]. However, only a subset of TNBC patients responds favorably to this treatment [64, 65]. Therefore, the identification of biomarkers capable of predicting treatment response is of substantial clinical importance for selecting patients most likely to benefit from ICIs. Through the application of TIDE, we demonstrated that the FDCSP effectively discriminates between responders and non-responders to ICB treatment. These findings suggest that FDCSP could serve as a novel candidate biomarker for predicting immunotherapy response.

## Conclusion

In conclusion, we conducted comprehensive profiling of both non-TNBC and TNBC tissues, employing an integrated multi-omics approach. Our investigation led to the identification of a unique FDCSP gene in TNBC, the characterization of its TNBC-specific FDCSP high expressed basal cells, and the elucidation of critical cellular interactions of FDCSP high expressed basal cells within the tumor microenvironment. These findings provide novel mechanistic insights into the molecular and cellular processes driving the malignant progression of TNBC, offering potential targets for therapeutic intervention and early detection strategies.

## Data Availability

The original contributions presented in the study are included in the article/Supplementary Material, further inquiries can be directed to the corresponding author.

## Footnotes

1
https://www.cancer.gov/ccg/research/genome-sequencing/tcga

2
https://www.ncbi.nlm.nih.gov/gds

3
https://www.10xgenomics.com/datasets

4
http://www.bioinformatics.com.cn/static/others/jvenn/index.html

5
https://cn.string-db.org/

6
http://gepia2.cancer-pku.cn/#index

7
http://care.dfci.harvard.edu/

8
https://github.com/XinYaLu9696/TNBC/tree/main

## Supplementary material

SUPPLEMENTARY TABLE S1 The expression of cell lines from CCLE databases.

SUPPLEMENTARY TABLE S2 M1 and M2 polarization-related genes of macrophages.

SUPPLEMENTARY TABLE S3 The relationship between FDCSP expression and drug efficacy in the CARE database.

SUPPLEMENTARY FIGURE S1 Common DEGs associated with TNBC identified in TCGA and GEO. **(A)** PPI network for G1, generated using STRING. **(B)** PPI network for G2, generated using STRING. **(C)** Volcano plot illustrating DEGs identified in TCGA data by comparing TNBC to non-TNBC. **(D)** Volcano plot illustrating DEGs identified in GSE76275 data by comparing TNBC to non-TNBC. **(E)** Venn diagram showing the overlap of up-regulated DEGs. **(F)** Venn diagram showing the overlap of down-regulated DEGs. **(G)** GO analysis of co-up-regulated DEGs and co-down-regulated DEGs. **(H)** KEGG analysis of co-up-regulated DEGs and co-down-regulated DEGs.

SUPPLEMENTARY FIGURE S2 Correlation between FDCSP expression level and TNM stage of breast cancer. **(A)** Box plot showing the expression levels of FDCSP in T1-4 stages of TNBC (T describing the size of the tumor and any spread of cancer into nearby tissue). **(B)** Expression levels of FDCSP in N1-4 stages of TNBC (N describing the spread of cancer to nearby lymph nodes). **(C)** Expression levels of FDCSP in M0 and M1 stages of TNBC (M describing the metastasis). **(D)** Expression levels of FDCSP in T1-4 stages of non-TNBC. **(E)** Expression levels of FDCSP in N1-4 stages of non-TNBC. **(F)** Expression levels of FDCSP in M0 and M1 stages of non-TNBC. **(G)** IHC staining showing the expression of FDCSP in Norma tissues. *P*-values were determined using Wilcoxon tests. *: *P* < 0.05.

SUPPLEMENTARY FIGURE S3 Distribution of single cells across different breast cancer subtypes. **(A)** UMAP representation of scRNA-seq data from different groups, including samples from normal breast tissue, ER+, HER2+ breast cancer and TNBC. **(B)** Feature plot showing FDCSP expression of all scRNA-seq data from different groups. **(C)** UMAP representation showing the composition of epithelial subtypes in different groups.

SUPPLEMENTARY FIGURE S4 Distribution of immune cell subtypes. **(A)** UMAP representation showing the composition of immune subtypes. **(B)** Dot plot showing the expression of marker genes for each immune subtype. **(C)** Bar plot showing the percentage of each cell subtype. Colors correspond to those used in panel **(A)**. **(D)** UMAP representation showing the composition of macrophage subtypes. **(E)** Feature plot showing TGF-β1 expression in all macrophage subtypes. **(F)** Feature plot showing the expression of M1 signature gene sets in all macrophage subtypes. **(G)** Feature plot showing the expression of M2 signature gene sets in all macrophage subtypes. **(H)** Dot plots showing the average expression of known markers in indicated clusters.

SUPPLEMENTARY FIGURE S5 Expression pattern of EGFR in breast cancer. **(A)** EGFR expression in TCGA. *P*-values were determined using the Wilcox tests (****P* < 0.001). **(B)** Feature plot showing EGFR expression in epithelial subtypes. **(C)** Feature plot illustrating FDCSP (red) and EGFR (green) expression in epithelial subtypes. **(D)** Correlation of FDCSP and EGFR expression. A *P*-value <0.05 was considered statistically significant.
