# Supplementary figures and images for: Multi-omics analysis identifies the unique high-FDCSP basal cells in triple-negative breast cancer
Source: Exp Biol Med (Maywood). 2025 Sep 25;250:10632. doi: 10.3389/ebm.2025.10632 (PMC12507714; doi:10.3389/ebm.2025.10632)

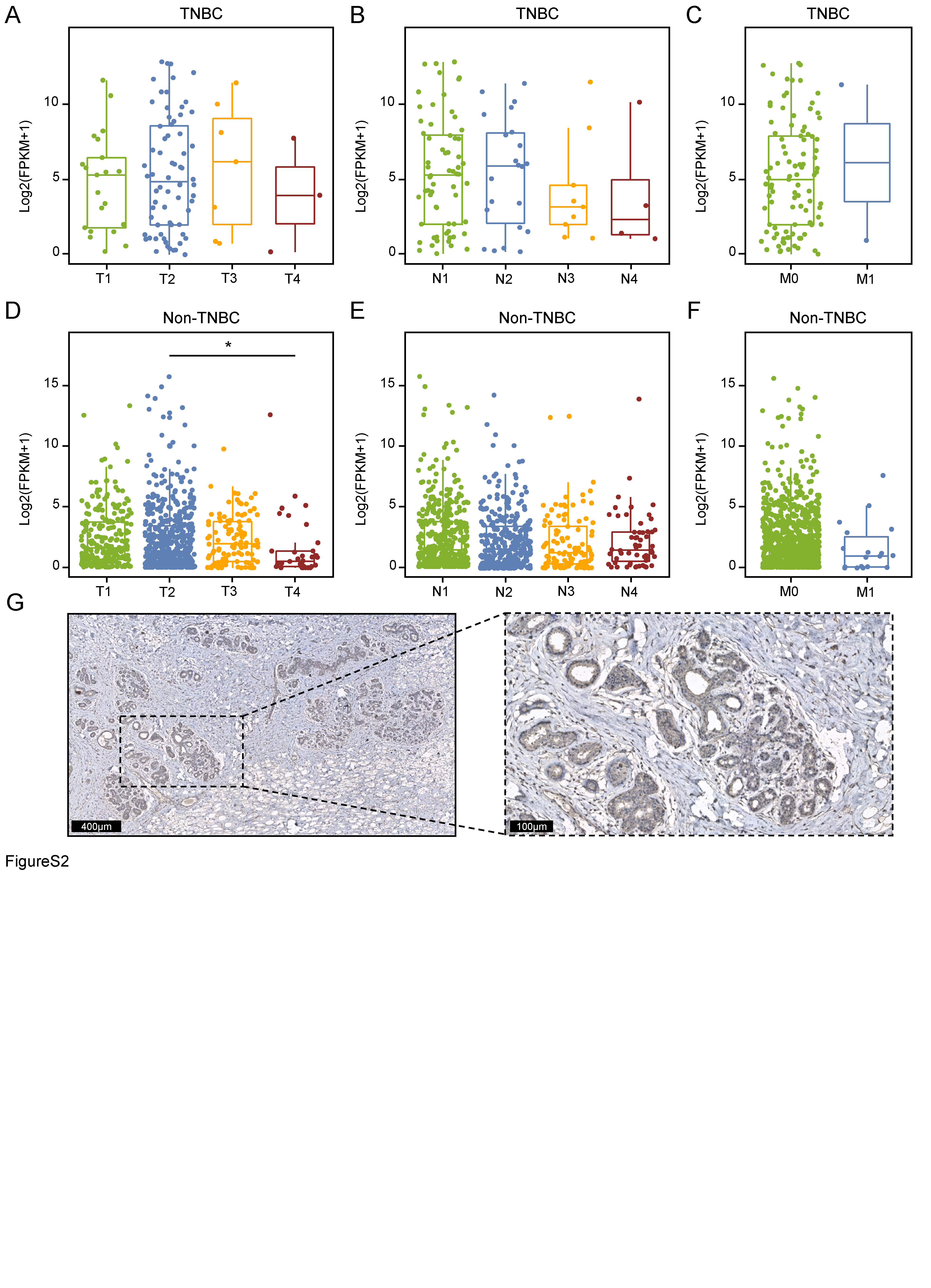

Supplement: Supplementary file 5 [file Image2.tif]
